# Welfare-improving enrichments greatly reduce hens’ startle responses, despite little change in judgment bias

**DOI:** 10.1038/s41598-019-48351-6

**Published:** 2019-08-15

**Authors:** Misha Ross, Anna Garland, Alexandra Harlander-Matauschek, Lindsey Kitchenham, Georgia Mason

**Affiliations:** Department of Animal Biosciences, University of Guelph, 50 Stone Road, Guelph, Ontario N1G2W1 Canada

**Keywords:** Animal behaviour, Psychology

## Abstract

Responses to ambiguous and aversive stimuli (e.g. via tests of judgment bias and measures of startle amplitude) can indicate mammals’ affective states. We hypothesised that such findings generalize to birds, and that these two responses co-vary (since both involve stimulus evaluation). To validate startle reflexes (involuntary responses to sudden aversive stimuli) and responses in a judgment bias task as indicators of avian affective state, we differentially housed hens with or without preferred enrichments assumed to improve mood (in a crossover design). To control for personality, we first measured hens’ baseline exploration levels. To infer judgment bias, control and enriched hens were trained to discriminate between white and dark grey cues (associated with reward and punishment, respectively), and then probed with intermediate shades of grey. For startle reflexes, forceplates assessed responses to a light flash. Judgment bias was only partially validated: Exploratory hens showed more ‘optimism’ when enriched, but Non-exploratory hens did not. Across all birds, however, startle amplitudes were dramatically reduced by enrichment (albeit more strongly in Exploratory subjects): the first evidence that avian startle is affectively modulated. Startle and judgment biases did not co-vary, suggesting different underlying mechanisms. Of the two measures, startle reflexes thus seem most sensitive to avian affective state.

## Introduction

Assessing animals’ affective states, and thence their welfare, requires well-validated indicators that reliably track affective valence, i.e. the positive and/or negative dimension of emotions and moods (e.g.^1,2^). Our aim was to test the validity of two potential indicators of affective state in chickens – judgment bias and startle amplitude – and to assess how they inter-relate.

In humans varying in affective state, judgment biases (sometimes called cognitive biases) that influence how ambiguous stimuli or situations are interpreted have been well studied. Data come from a host of diverse unconditioned tasks involving images, text, auditory stimuli and personal narratives; and as Mendl and colleagues summarize, these reveal that “people in negative states tend to judge ambiguous stimuli negatively … more readily attend to threatening stimuli and recall negative memories than people in positive mood states”^3^. Tests to measure similar judgment biases in non-human animals have been developed to assess their affective states^4–6^. Here, conditioned tasks are used: often Go/No go tasks where animals are first trained to perform an action to obtain a food reward in the presence of a specific discriminative stimulus (the DS+, e.g. a black cue), and to avoid performing this action to avoid punishment in the presence of a different discriminative stimulus in the same modality (the DS−, e.g. a white cue). Subjects’ responses to ambiguous stimuli (with properties intermediate between the DS+ and DS− cues; e.g. here shades of grey) are then measured as indications of their ‘optimism’ or ‘pessimism’, such that their tendencies to interpret an ambiguous cue as a DS+ or DS− indicate positive or negative affective states respectively. In one pioneering study^6,7^ rats were trained to associate high- and low-frequency sound tones respectively with reward and punishment, and then probed with ambiguous intermediate tones. Like humans in negative affective states, rats subjected to stressful housing conditions responded more ‘pessimistically’ to these ambiguous stimuli, treating such tones as if more likely to be a DS− than DS+.

In many similar subsequent studies, animals exposed to aversive treatments have been found to have negative judgment biases^5,6,8^; and some studies also show that preferred, presumed pleasurable, stimuli can induce positive biases (e.g.^9,10^). However, not all such studies find the expected effects^5,6^. This is perhaps unsurprising, since the same holds in the species for which judgment biases are best understood: humans. Thus tests for judgment bias are not always sensitive to humans’ affective state (leading to null results in e.g. some studies of people with pain, depression or anxiety^11–14^. Reasons for such false negative results include that findings can be influenced by the type of task used (e.g.^12,13,15^), and by subjects’ developmental stage^14^. In addition, processes like emotional regulation can even have effects that *oppose* the expected effects of judgement biases. For example, people with depression can be *poorer* at recalling pain-related words from a list than those without (rather than better, as judgment bias accounts would predict), perhaps due to a desire to avoid such words^11^; and higher anxiety scores can predict the expectation of *more* positive events (rather than fewer)^13^. Positive moods can also *increase* aversion to risk (rather reducing it, as bias accounts would predict), in subjects keen to protect and maintain their currently pleasant states (e.g.^15^). Thus even in humans, because of both extrinsic influences (e.g. task design) and intrinsic effects (e.g. age and emotional regulation), judgment bias tests do not infallibly reveal ‘optimism’ in positive affective states and ‘pessimism’ in negative affective states. To be valuable in animal welfare assessment, any new judgment bias task must thus first be validated as an indicator of that species’ affective state, rather than simply assumed to be one. This was therefore one of our aims for hens.

In contrast to judgment bias, another potential measure of stimulus evaluation – the startle reflex – has been as yet little used in animal welfare research. Startle reflexes are rapid, non-directional muscular responses, typically lasting under 450 ms, involving eye blinks, head movements and whole body jerks (e.g.^16–19^). They are elicited by intense stimuli that are unexpected, sudden, and reported as aversive, even – as is typical for laboratory studies – when actually harmless (e.g., a sudden sound, bright flash of light, or rapid air puff to the face or body^17,18,20,21^; and they may serve as defensive mechanisms^20,22^. The likelihood and amplitude of startle reflexes can be influenced by background affective state (e.g.^19,21^: at least in mammals (including humans, non-human primates, rodents and cats), negative states such as fear and anxiety typically increase them^20^ while positive states (e.g. induced by pleasant images, pleasant odours, or monetary prizes) often decrease them (e.g.^19,21^; also^23–32^ [all related to picture viewing];^33^ [odour]; and^34^ [money prizes]. Startle amplitudes (individual-level responses) and startle magnitudes (group level averages, including non-responses) are therefore widely used as indicators of affective state in human research and behavioural neuroscience (e.g.^20,21,35^).

However, just like judgment bias, startle is not an infallible indicator of affective state. For example, in humans the negative states of insomnia^36^ and disgust (e.g.^37^) often fail to potentiate startle; while in rats, inducing lassitude and fever with LPS reduces rather than enhances startle magnitudes, despite this being a negative state^38^. Furthermore, in humans, positive affect manipulations do not always successfully reduce startle reflexes (e.g.^20^); indeed anticipating rewarding events may even *potentiate* startle (e.g.^32^). Such effects may reflect how much readiness for physical action each state induces^39^. Exploring how known affective states influence startle is therefore important if this response is ever to become useful in welfare assessment, and especially so for non-mammals: birds have startle reflexes homologous with those of mammals^40,41^, but whether these reflexes are subject to similar affective modulation had not been investigated. Assessing the impact of affective state changes on the avian startle reflex, using laying hens as a model, was therefore our second aim.

Our third and final aim was to assess whether negative judgment biases predict enhanced startle. After all, many human conditions characterized by enhanced startle (e.g. anxiety and PTSD) also involve negative judgment bias (e.g.^42,43^). Furthermore, Mendl and colleagues argue that “mood-dependent cognitive biases are likely to influence appraisals of emotion-inducing stimuli …. (e.g. negative cognitive biases may underlie a negative appraisal of an event)”^3^: relevant because startle reflexes do appear to involve the appraisal of emotion-inducing stimuli. Thus startle amplitudes are increased, not only by pre-existing affective states, but also when the eliciting stimulus is perceived as more aversive^44^, including when the stimulus is objectively more intense, sudden (i.e. has a more rapid ‘rise time’), or unexpected (i.e. with no warning or opportunities to habituate)^26,45–47^: all factors that make it subjectively more unpleasant^48–50^. Thus faced with objectively the same startling stimulus, negative affective states typically cause subjects to react as if evaluating it as relatively more intense, sudden and aversive; while positive affective states, in contrast, often have the opposite effect, causing subjects to react as if evaluating the stimulus as relatively *less* intense, sudden and aversive^19,22,51^. Since both judgment bias and startle reflexes thus involve affectively modulated stimulus evaluation, we aimed to see if these two responses correlate (perhaps with negative judgment biases playing a predictive role^3^).

To test these three hypotheses, our experimental design was based on the following principles. First, as our treatment, we manipulated affective state with the aim of making it more positive. This was partly on ethical grounds, but also because while understanding of positive welfare is a growing research area^2^, whether judgment bias tests are sensitive to positive affect is less well established compared to for negative affect^8,52^. Second, we used enriched housing that not only contained resources *a priori* chosen to be positively reinforcing for hens^53^, but which was confirmed as being preferred by our subjects via *in situ* preference tests (*cf*.^54^). This gave us confidence that our treatment did induce positive affect relative to our Control group. From Mendl *et al*., for example, we would argue that such low threat situations in which rewards are reliably present should induce a “positive state of contentment or satisfaction”^3^ (see also^55^) at least relative to controls; and human data generally support this^56^. Third, we followed previous studies in assessing and controlling for personality in our subjects; this both allows assessment of the generality of effects across different temperaments, and improves test sensitivity by factoring out the noise that individual variation would otherwise add^57,58^. Our hens were therefore first profiled using a series of arena and novel object tests. Fourth and finally, after testing hens who had been differentially raised since the pullet phase (puberty) in Control or Enriched conditions for seven weeks, we followed Bateson and Matheson’s starling work^59,60^ in reversing the birds’ housing and re-tested them soon afterwards. This allowed us to determine if enrichments’ effects on judgment bias and startle reflex were quickly reversible, as would be expected if caused by affective state, as well as enabling powerful within-subject statistical approaches.

Overall, our hypotheses and predictions were thus as follows: if judgment bias and startle amplitude are valid indicators of affective state, then providing hens with access to preferred, enriched housing (which should increase their positive affect), will bias their evaluation of ambiguous conditioned stimuli and aversive (but non-harmful) unconditioned stimuli, such that compared to Control hens, Enriched hens will show more ‘optimistic’ responses to ambiguous probes in judgment bias tests, and also reduced startle amplitudes to sudden stimuli. If these two responses share common underlying mechanisms (e.g. those involved in the judgment bias test influence responses to unconditioned emotional stimuli), then this predicts that these two affect-modulated evaluation responses will co-vary.

## Methods

### Ethics statement

This work was approved by the University of Guelph Animal Care Committee, Animal Use Protocol #3763. Methods were carried out in accordance with relevant guidelines and regulations. Hens were monitored daily to ensure they were physically healthy.

### Subjects

Twenty-four ISA Brown hens (Hendrix Genetics, Boxmeer, the Netherlands) were used, selected from a larger sample of 96: two separate cohorts of 48 hens (Cohort 1 kept in Summer 2017, and Cohort 2 in Fall 2017), each obtained at 18 weeks of age from a different commercial supplier. Upon arriving at the research barn, each hen was fitted with a leg ring for individual identification and spent one month habituating to the new environment while housed in groups of 12 in four floor pens provisioned with perches, nest boxes, softwood shavings and *ad libitum* feed (Purinature Layena® crumble) and water, and kept on a 15: 9 light: dark cycle (with a 15-minute artificial dusk provided in the evening) at 20 °C.

### Personality testing, subject selection and allocation to treatment

During each habituation period, 12 hens per cohort were chosen for testing based on divergent levels of exploratory behaviour, assessed with methods that followed Asher *et al*.^57^. Three tests were conducted over three consecutive days: an arena test on Day One; and novel object (NO) tests on Days Two and Three. Tests were conducted in a plywood arena measuring 1.2 m × 1.2 m × 1.2 m, with a plastic mesh ceiling and corrugated white plastic floor marked with 16 equal squares (each 30 cm × 30 cm; see^53^. Prior to testing, an entire pen of hens was put in a 0.75 m^2^ metal dog crate (bedded with softwood shavings and containing a 4-liter fountain drinker). Hens were then tested in a predetermined random order. Behaviour was filmed using a video camera mounted overhead (Sony CX405 Handycam®). For the arena test, hens were individually placed in the center of the arena for three minutes, and the number of squares they entered during this period was counted. For each NO test, hens were individually placed in a start box for one minute measuring 33 cm long × 28 cm wide × 56 cm high, positioned on the opposite side of the arena from the NO (see^57^). A guillotine door was opened, and the hen allowed five minutes to exit the start box and explore the NO (either a white bucket [NO test 1] or an orange traffic cone [NO test 2]). Each hen’s latency to exit the start box and approach the NO was assessed, the hen being scored as contacting the NO if any part of her body was visibly touching it, or if any part of her body overlapped with the object as viewed from the overhead camera.

Within each cohort, hens were assigned a rank for each test. For the arena test, ranks were assigned based on the number of squares hens entered (more squares = higher rank). For NO tests, ranks were assigned based on latencies to exit the start box and approach the NO (shorter latencies = higher rank). In both cohorts, hens’ ranks across tests were positively associated (across the two NO tests, Spearman’s ρ ≥ 0.74, N = 48, P < 0.001; and across each NO test and the arena test: ρ ≥ 0.51, N = 48, P < 0.001). An aggregated rank was therefore assigned to each hen per cohort by combining her three ranks. Six of the highest-ranking hens (‘Exploratory’), and six of the lowest ranking (‘Non-exploratory’) were selected per cohort as subjects for this experiment (totaling 12 Exploratory and 12 Non-exploratory hens across both cohorts). At 22 weeks of age, these hens were systematically assigned to Enriched and Control housing so that personality was balanced across housing conditions, in a factorial design (Table 1). These were allocated to separate pens to ensure statistical independence, with assignment to pen pseudo-randomized to ensure a balanced design. Each experimental hen was then grouped with three other hens familiar from the habituation period (also pseudo-randomly selected), so that each pen contained four hens.

**Table 1:** Allocation of hens to housing treatment according to personality.

Cohort 1	6 Enriched groups	3 groups with an Exploratory hen, 3 groups with a Non-exploratory hen
	6 Control groups	3 groups with an Exploratory hen, 3 groups with a Non-exploratory hen
Cohort 2	6 Enriched groups	3 groups with an Exploratory hen, 3 groups with a Non-exploratory hen
	6 Control groups	3 groups with an Exploratory hen, 3 groups with a Non-exploratory hen

### Differential housing

Each group of Control hens was housed in a 1.5 m^2^ plywood enclosure measuring 1.2 m × 1.2 m × 1.2 m. Each Control pen contained a nest box, a perch, softwood shavings and *ad libitum* feed (Purinature Layena® crumble) and water. Each group of Enriched hens was provided a larger area (9 m^2^), also bedded with softwood shavings but with added features making it more varied and spatially complex: perches and platforms at various heights, foraging opportunities, and an assortment of enrichments including sand and peat for dustbathing. All features were chosen *a priori* as likely to be preferred^53^, but to confirm this, each Enriched pen was also attached to a ‘Proxy’ Control pen: a mock-up of a Control pen used to test hens’ environmental preferences *in situ*. Feed and water were provided *ad libitum* on the border of the Enriched pen and the Proxy Control pen, so that Enriched hens could access these from either environment. To check that Enriched housing was indeed preferred, Enriched hens’ locations were recorded during the entire 60-day housing period. A camera (RY-208C Mini CMOS 420TVL) was mounted over each Proxy Control pen, and recordings used for instantaneous scan sampling at 30-minute intervals between 8:00 to 19:00. Longterm *in situ* preference data were used to calculate Hens’ relative use of Enriched and Proxy Control areas after controlling for the difference in area between the two environments (since hens would spend six times [9/1.5] as much time in the larger than the smaller pen by chance alone).

### Experimental timeline

After the move to the housing treatments (Day 0), hens were trained on a judgment bias task (Days 0–33), had their startle reflexes tested twice (Days 35 & 37), and then received five judgment bias test sessions (Days 38–42). After 48 days, housing was switched so that Enriched hens now occupied Control pens, and *vice versa*, for an additional 12 days. Hens then received a second set of judgment bias tests (Days 53–57) and two more startle tests (Day 58 and 60): see Table S1 in the Supporting Material.

### Judgment bias training and testing

#### The apparatus

**Figure 1:**
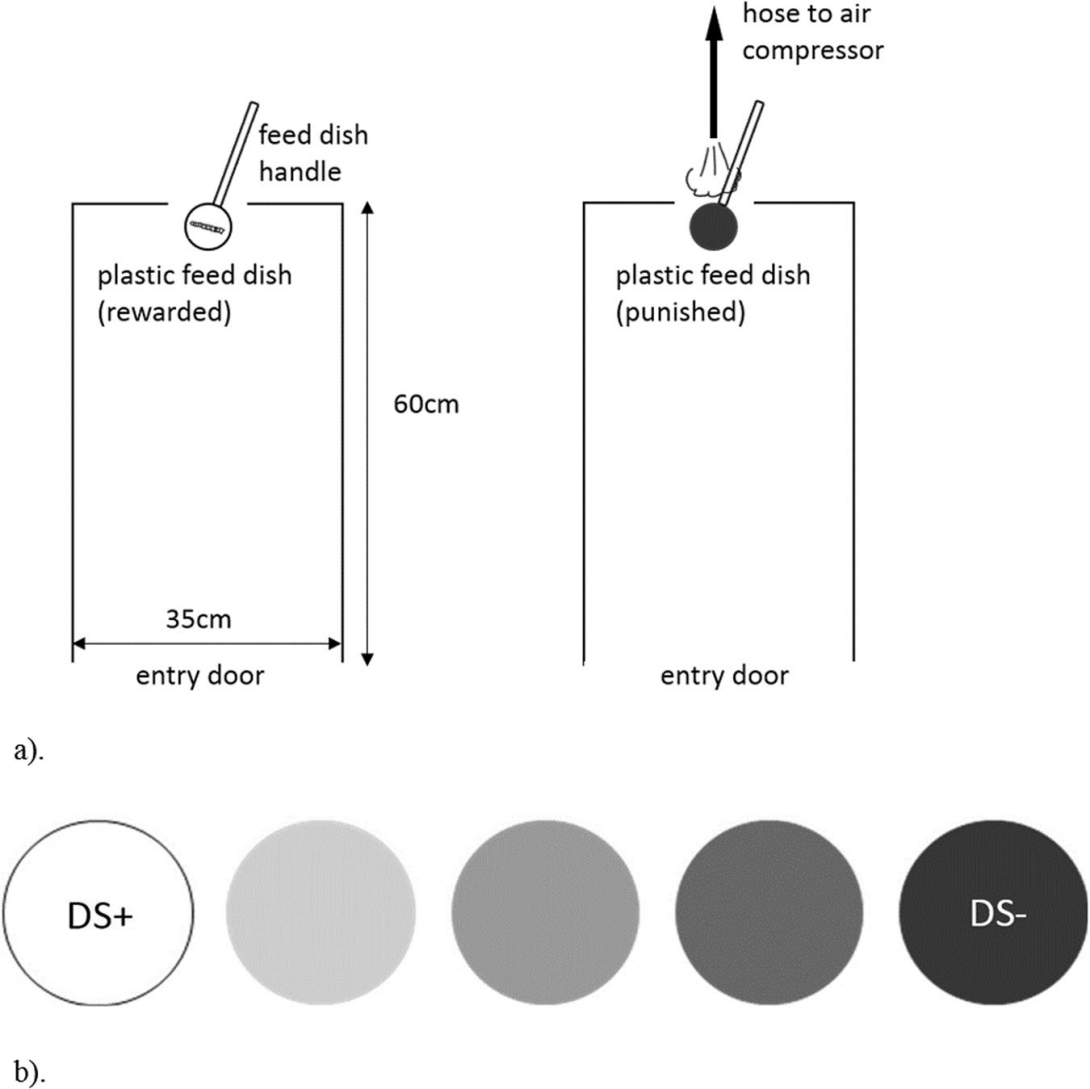
The judgment bias apparatus. **(a)** Overhead view of the judgment bias chamber as using in training as well as testing. During training, when presented with a white lid, hens were rewarded with a mealworm (left), and when presented with an 80% grey lid they were punished with an air puff (right). (**b)** The five different lid shades used during judgment bias testing. White (DS+ training cue), 20% grey (probe), 40% grey (probe), 60% grey (probe) and 80% grey (DS− training cue).

Cue discrimination training and judgment bias testing were conducted in a plywood chamber (Fig. 1a), with a corrugated plastic floor and metal mesh ceiling. This measured 60 cm long × 35 cm wide × 45 cm high, with a 35 cm wide × 30 cm high door that was closed during training and testing. An overhead camera (RY-208C Mini CMOS 420TVL) allowed hens to be observed remotely (to avoid influencing their behaviour).

Hens were trained to discriminate between two cues: a white disc-shaped plastic lid (DS+) which, if displaced, exposed a mealworm located underneath (US+) and an 80% grey disc shaped plastic lid (DS−) which, if displaced, resulted in punishment via an air puff to the face (US−). The protocol was modified from methodology previously used in starlings^59^. Training consisted of habituation and pretraining stages, and then a cue discrimination training stage.

#### Habituation and pre-training (day 0–7)

Hens were individually removed from their pen and placed inside the chamber where they were given 10 mealworms (a highly preferred reward for chickens), individually placed on the plastic floor. Next, inside the chamber, hens were offered mealworms presented individually in an uncovered round plastic food dish (6 cm diameter × 2.5 cm high), fastened to a handle so it could be inserted through a 10 cm wide × 3 cm high opening located in the chamber wall at chamber floor level (Fig. 1a). Hens’ responses were monitored with the camera, and when they consumed the mealworm, the dish was removed and refilled. For each session, each hen received 10 dish presentations (trials) of one mealworm each. This was repeated until hens were reliably eating all 10 mealworms, which typically occurred during the first session. Next, hens were exposed to the same dish presentations (10 trials of 1 mealworm each), but the dish was partially covered with a white lid (made of corrugated plastic of slightly larger diameter than the dish, covered with white paper (20 lb, 92 bright). This lid was attached to the dish handle with a thin nylon line, so it could be retrieved after being displaced. For the first training session, the lid covered 1/2 of the dish, after which it was placed to successively cover more of the dish (1/3 to 2/3 to fully covered). These sessions were continued until hens were reliably eating the mealworms when the dish was fully covered (criterion set at 100% success in 10 trials across 3 consecutive sessions). All hens met this criterion within 10 sessions.

#### Cue discrimination training (day 8 to 33)

Hens were next introduced to the punished 80% grey lid, identical to the white lid but covered with paper (laser printed with CMYK colour code 0,0,0,80). Hens were trained to avoid displacing the grey lid, otherwise they were punished with an air puff (50–80 psi lasting <1 s) directed towards the head using a regulated air compressor with a blow gun and 1.5 mm diameter nozzle attachment (Campbell Hausfeld, Harrison, Ohio). Its duration was manually controlled with the blow gun valve located outside of the test chamber. An air puff has been validated as an aversive yet harmless punishment for chickens^61^, and successfully used in other cue discrimination training protocols for this species^62,63^. For the initial punished session two 80% grey lids were introduced, and this was gradually increased until sessions contained equal numbers of rewarded and punished lids (20 lids total: 10 DS+, 10 DS−). All hens met the criterion for cue discrimination training within 19 sessions.

Throughout training, hens were presented with either lid type for a maximum of 10 s. If a lid was displaced, the dish was removed from the test chamber after the hen consumed the mealworm or received the air puff. The inter-trial interval between lid presentations was 15 s. Lid order was changed every training day and pseudo-randomized so that hens did not receive more than three consecutive DS+ or DS− cues. Two to five training sessions per week were carried out for each hen over a period of approximately five weeks. Criterion for successful training was set at ≥80% successful responses for each of the DS+ and DS− cue types for three consecutive sessions. All 24 hens successfully reached criterion and were graduated to the judgment bias testing phase (after startle assessment: see below and Table S1).

#### Judgment bias test 1 (days 38–42)

Hens’ responses to ambiguous probes, consisting of intermediate lid shades, were now measured. Five test sessions were carried out between the late morning and early afternoon over five consecutive days, each hen being tested once daily. As with the training sessions, hens were individually picked up from their home pen, carried to the test chamber and gently placed inside. The chamber door was immediately closed to contain the hen after which testing began within 30 s by inserting the first cue into the chamber. The test sessions, each lasting approximately nine minutes, consisted of 26 trials: 10 DS+ and 10 DS− cues (to keep the opportunity for reward acquisition consistent with training^62^; and two presentations each of three different ambiguous probe cues interspersed between the trained cues (2 × 20% grey, 2 × 40% grey and 2 × 60% grey: CMYK colour codes 0,0,0,20; 0,0,0,40; and 0,0,0,60 respectively) (Fig. 1b). In keeping with past studies e.g.^62^ during testing, the DS+ and DS− cues were reinforced and punished respectively, to avoid extinction of cue discrimination, while the probe cues were unreinforced to minimize any learned associations with positive or negative outcomes. As in training, lids were presented for a maximum of 10 s and the inter-trial interval was 15 s for both trained cues and ambiguous probes. The order of lid presentation was pseudo-randomized to avoid more than three consecutive DS+ or DS− cues, or more than two consecutive probe cues. The order of lid presentation was kept consistent between hens on each test day and changed between testing days. During testing, a mealworm was attached to the underside of each of unrewarded lid to ensure that the probe lids were truly ambiguous (and to prevent hens from using odour to identify when a mealworm was accessibly present in the dish). Out of the 24 successfully trained hens, only 23 were tested because one hen sustained an unrelated injury and was euthanized by captive bolt (Zephyr EXL, Bock Industries) prior to testing. For each cue shade, the number of displaced lids was recorded in real time using the computer monitor. A lid was counted as displaced if it was moved enough that the hen was able to fit her beak into the dish.

#### Judgment bias test 2 (days 53–57)

Following the housing switch, hens underwent an additional five judgment bias test sessions using methods identical to Judgment Bias Test 1. One session was conducted per day such that hens were tested between days five and nine following the housing switch. The post housing-switch habituation period was similar to that of Bateson and Matheson’s (2007)^59^ study of judgment bias in starlings, which detected a housing effect in the short term aftermath of a housing change.

### Startle reflex measurement

Startle reflexes were measured in a plywood chamber measuring 96 cm long × 96 cm wide × 60 cm high. A 35 cm long × 35 cm wide × 3 mm thick aluminum plate covered with a sanded, non-slip roofing membrane (RESISTO Self-Adhesive Roofing Underlay) was centered inside it. This aluminum plate was mounted using Scotch® double-sided carpet tape to a 15 cm × 15 cm custom-made force plate (as described in^64^) to allow for measurement of ground reaction forces from any location on it. Four 30 cm-tall steel wire grid fences were used to confine hens to the aluminum plate. To prevent injury, these were suspended with strings from the chamber ceiling so that they would give way if a hen bumped into them.

**Figure 2:**
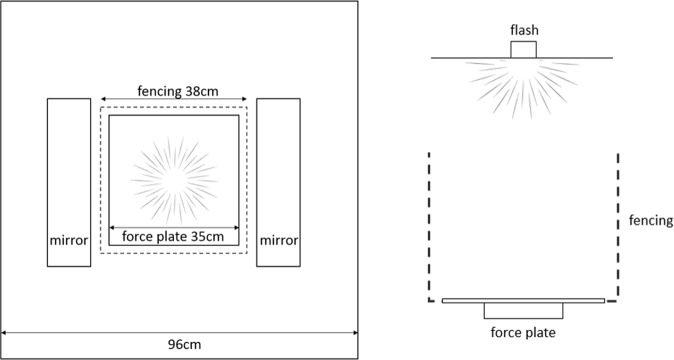
Overhead (left) and side (right) view of the startle chamber. Hens were individually placed on the aluminum force plate in the dark chamber, and confined with wire fencing. Flashes were mounted on the chamber ceiling; when they discharged, their light was reflected off the mirrors on the chamber floor. The force plate recorded forces along x, y and z-axes.

Following startle work on pigeons (e.g.^40^), a visual stimulus was used. The startle stimulus thus consisted of two simultaneously discharged Nikon Speedlight SB-20 xenon camera flashes set to full aperture (intensity), mounted to the chamber ceiling. Each flash had a duration of 0.8 ms and a rise time (latency from first light emission to full intensity) of less than 0.005 ms (Nikon SB-20 Instruction Manual). Based on previous pilots^53^, the flash was made more startling by keeping the ambient light level inside the chamber low (approx. 1 lux) and by reflecting their light off the floor using two 12 cm × 45 cm mirrors (Fig. 2). To mask abrupt extraneous sounds that could cause prepulse inhibition (cf. e.g.^65^), an electric fan (7-inch Honeywell Super Tech Force High Performance Fan) was kept running on the floor adjacent to the chamber throughout testing (mimicking standard procedure for rats and humans, where white noise is often supplied as a background sound).

#### Startle reflex test 1 (days 35 and 37)

Hens were startled twice, always in the afternoon and interspersed by one rest day. Hens were individually placed in the startle chamber, and after 30 s the camera flashes were discharged. These were synchronized to the force plate using a 555-timer circuit, connected to the force plate data collection software through the computer. Thirty seconds after the flash, hen mass was recorded. The hen was then removed from the chamber and returned to her pen-mates (spending a total of 1 minute in the chamber).

The datafile produced by the force plate software was used as a reference to identify the time of flash, which occurred 30 s (±150 ms) after the force plate software began logging data (the 300 ms range accommodating 0.5% error in the timer circuitry). A three-dimensional resultant vector (combining x, y and z-axis forces) was then calculated for each hen and used to quantify peak startle amplitude (i.e. the maximum three-dimensional force exerted into the force plate) during the 300 ms period when the light flash was discharged, with an additional 100 ms allowance on the later edge of the range to accommodate reaction time (cf. e.g.^18^). Scoring of the resultant vectors, which we previously determined to have high interobserver reliability (Pearson’s r = 0.99, N = 16), was conducted blind to treatment (see^53^ for more details).

#### Startle reflex test 2 (days 58 and 60)

Startle reflexes were measured two additional times following the housing switch, using methods identical to Startle Reflex Test 1.

### Judgment bias

For the judgment bias test, we followed Brilot *et al*. (2012)^66^ and discarded sessions where a hen did not meet the training criterion (≥80% successful responses for each of the DS+ or DS− cues), so that inaccurate cue discrimination did not contribute to hens’ judgment bias scores; e.g., if a hen flipped too many punished cues, then any ambiguous lids flipped may be caused by poor cue discrimination instead of ‘optimistic’ responding. In Judgment Bias Test 1, 6 out of 115 test sessions were discarded due to hens failing to meet criterion; while in Test 2, 13 out of 115 sessions were discarded, including one hen who never met criterion during any session and was thus omitted from the analysis.

Data from probe trails were analyzed using a generalized linear mixed model (PROC GLIMMIX), following Gygax (2014)^67^. The response variable (whether hens flipped the ambiguous cue in each trial) was analyzed as a binary response. The fixed factors were: housing (Control vs. Enriched); personality (Exploratory vs. Non-exploratory); period (before and after the housing switch); cohort (included as a fixed effect because the different cohorts were obtained at different times of year from different suppliers); session; and cue shade (20, 40 and 60% grey, which was randomized at the session level for all hens). The housing*personality interaction was included to test our prediction that hens would be differentially affected by housing depending on their temperament^57^. Several additional interactions relevant for interpretation were also included: the two and three-way interactions between housing, personality and cue shade (to determine if the data should be split by cue shade to investigate housing or personality effects); and the two and three-way interactions between housing, personality and period (to determine if any carryover effects were present after housing was switched, the absence of which would allow us to interpret the results of the full cross-over experiment). Finally, due to the possibility that hens’ responses to the unreinforced ambiguous probes could extinguish over time, we included the two and three-way interactions between housing, personality, and test session. A lack of interaction between these factors would indicate that neither housing nor personality influence the rate of response extinction, and would thus warrant including all test sessions in the analysis. For the random effect, hen ID was nested in personality and cohort. In a separate random statement, test session was also nested in period, hen ID, housing and personality to ensure these fixed effects were estimated with the correct degrees of freedom (using the Kenward-Roger’s approach).

In addition, to check if differences in hens’ responses to the rewarded and punished training cues (DS+ and DS−) may have caused any differences in ambiguous cue responses, we repeated the same analysis except substituting the trained training cues for the ambiguous probe cues.

### Startle amplitude

For the startle analysis, the dependent variable was the maximum force exerted into the force plate, measured in millinewtons, which was averaged for each hen across the two test days for each of Startle Reflex Test 1 (Startles 1 & 2) and Startle Reflex Test 2 (Startles 3 & 4). To investigate effects of housing and hens’ personalities, the data were first combined across housing periods using a full factorial repeated measures general linear models involving the fixed factors: housing (Control vs. Enriched); personality (Exploratory vs. Non-exploratory); period (before and after housing the housing switch); and cohort (Summer vs. Fall). The model also included hen mass as a covariate, since this can be an important determinant of force^68^. Analyses revealed a significant period*housing interaction (F_1,35_ = 4.75, P = 0.036), indicating that the magnitude of the effect was different across housing periods and so complicating interpretation of within-subject comparisons. Therefore, the data were split by period, and between-subject comparisons analysed using two general linear models: one comparing Control and Enriched hens prior to the housing switch; and a second comparing Control and Enriched hens after housing was reversed. Normality of residuals was checked for all models using Shapiro-Wilk tests (W > 0.92, P > 0.09), and homogeneity checked by visually examining residual plots.

### Relationship between judgment bias and startle amplitude

To investigate whether startle amplitudes reflect judgment bias, we tested if hens’ propensities to flip ambiguous lids predicted their startle amplitudes using JMP (v. 13, SAS Institute, 2016). The proportion of lids flipped was calculated by pooling together the 20, 40 and 60% grey ambiguous cue shades, producing two values per hen: one value across all five test sessions prior to the housing switch (pre-switch) and one value across all five test sessions following the housing switch (post-switch). These proportions were then added as a covariate in the same general linear model used for analyzing housing and period effects on hens’ startle responses, the only other differences being that startle amplitudes were box cox transformed to meet model assumptions, and interactions with the judgment bias proportions were added to the model to reveal whether any releationship between the two measures was manifest only in some subgroups.

### Sample size calculations

Finally, to compare the sensitivity of the judgment bias and startle tests, we estimated the sample sizes required to detect the housing (mood) effect for between-subject comparisons using a simple between-subject t-test. For this we used pre-housing switch data (and set α to be 0.05 and power as 0.8, for a two-tailed test). For judgment bias, we again used the proportion of ambiguous lids that hens flipped (performing an arcsine square root transformation to achieve normality). For the startle data we used hens’ average amplitudes (the mean of Startles 1 and 2), log-transformed to achieve normality. Next, we calculated the mean difference between housing treatments, and pooled standard deviation, before inputting the effect size values (see Supporting Material) into several sample size calculators for independent sample t-tests (e.g. http://www.sample-size.net/sample-size-means/; all calculators used gave the same results). Required sample sizes to detect housing were estimated effects both without controlling for personality (Exploratory and Non-exploratory hens pooled); and after splitting the data by personality.

## Results

### Housing preferences

When hens were housed in the enriched pens, location data pooled across housing periods showed that they occupied the Proxy Control pen accessible to them significantly less than expected by chance. This includes hens who were housed in Enriched pens first, and then switched to Control housing, and *vice versa* (S_23_ = 150, P < 0.001; Median, Q1, Q3 = 0.047, 0.028, 0.096 versus 0.953, 0.904, 0.972, Proxy Control and Enriched respectively). Thus the Enriched environments were indeed preferable to Control environments.

### Judgment bias

**Figure 3:**
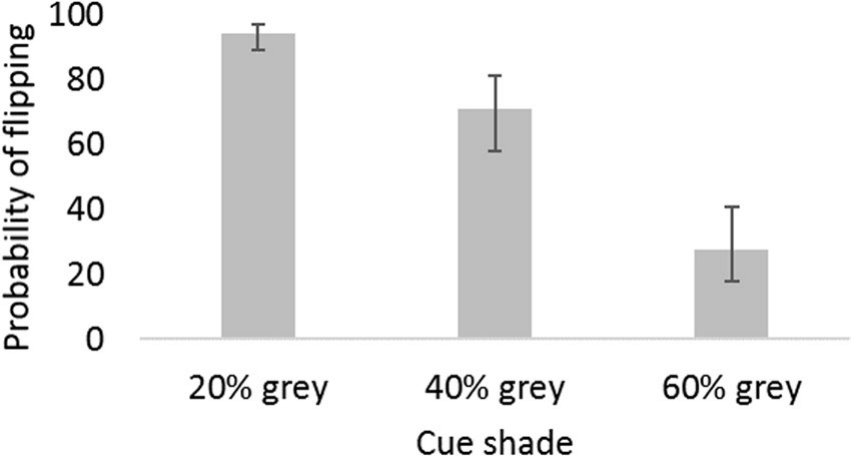
Hens’ responses to the unreinforced ambiguous cues. Hens’ responses to the unreinforced ambiguous cues corresponded to their resemblance to the training cues: white rewarded cues (which hens were trained to flip) and 80% grey punished cues (which hens were trained to avoid). Bars show the back-transformed Least Square Mean (LSM) probabilities of flipping the ambiguous lids and 95% confidence intervals. (LSM values partial out the effects of cohort, period, personality and interactive effects).

As expected, there was a significant negative relationship between cue shade darkness and the proportion of lids the hens displaced (F_2,1215_ = 131.7, P < 0.001), with hens’ responses graded according to the ambiguous cues’ resemblance to the trained training cues. See Fig. 3 Neither housing or personality interacted with cue shade (P > 0.36), so in subsequent models we did not separate the data based on cue shade (instead looking at housing and personality effects across all ambiguous shades pooled: cf.^67^.

The effect of period on judgment bias was significant (F_1,154_ = 8.17, P = 0.005) but did not interact with housing (P = 0.71) indicating that any influence of previous housing condition did not carry over to differentially influence hens’ behaviour after the environments were switched. This thus validated both pre housing switch and post housing switch periods being analyzed together as a full crossover experiment. Test session was also significant (F_4,150.9_ = 3.51, P = 0.008): proportion of cues flipped in session one was higher than sessions two, three and five; and was higher in session three than session five. However, session did not interact with housing or personality (nor was the three-way interaction significant) (P > 0.50), indicating that this decrease of response over repeated sessions did not differentially affect the main predictors. The effect of cohort was also not significant (P = 0.63).

**Figure 4:**
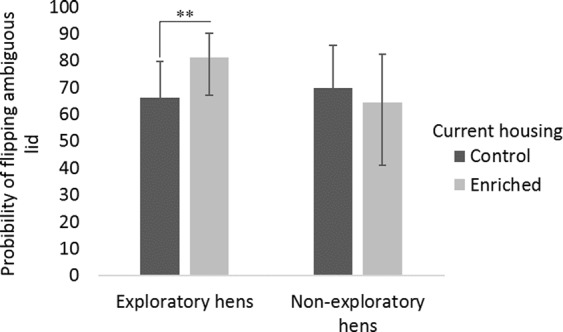
Housing and personality interact to affect judgment bias. Exploratory hens showed more optimistic responses when housed with preferred enrichments, but there was no housing effect for Non-exploratoy hens. Bars show the back-transformed least square mean (LSM) probabilities of flipping the unreinforced ambiguous lids and 95% confidence intervals. (LSM values partial out the effects of cohort, period and interactions). The legend denotes hens’ current housing during the time of testing. **P < 0.05.

Neither the main effect of housing (F_1,191.5_ = 1.67, P = 0.20) nor personality (F_1,15.09_ = 0.33, P = 0.58) was significant, but there was a significant interaction between housing and personality (F_1,191.4_ = 5.78, P = 0.017). This proved to be because Exploratory hens in Enriched housing flipped a higher proportion of ambiguous lids than Exploratory hens in Control housing (F_1,90.2_ = 5.73, P = 0.019; 81.23 (67.20, 90.13) vs. 66.29 (49.44, 79.82)) (back transformed LSMs and 95% confidence intervals for Enriched and Control hens respectively); while for Non-exploratory hens, in contrast, the effect of housing did not even approach significance (F_1, 103.8_ = 0.79, P = 0.38 (Fig. 4). Analysis of influence on responses to training cues confirmed that neither housing, personality, or the housing*personality interaction significantly predicted the proportion of punished or rewarded cues that were flipped (P > 0.1).

### Startle reflex amplitudes

**Figure 5:**
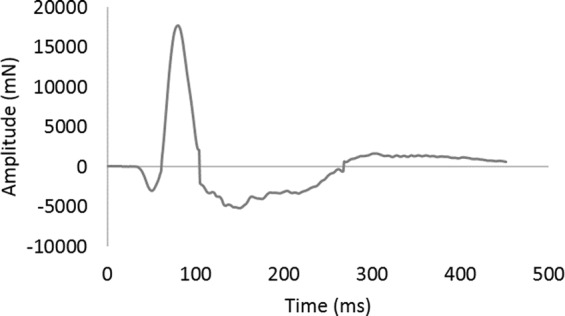
A typical startle reflex trace. t = 0 is the approximate time of the flash (for a bird with a peak amplitude of around 18,000 mN).

**Figure 6:**
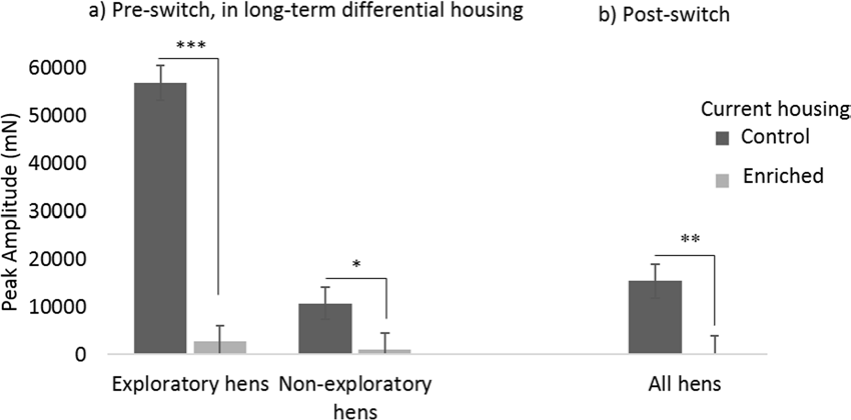
Housing affects startle. Enriched hens’ startle responses were greatly reduced compared to Control hens. Bar graphs show Least Square Means (LSMs) ± SEs of startle reflex amplitudes in millinewtons for hens housed long-term in Control and Enriched housing (pre-switch: **a**) and following the housing reversal (post-switch: **b**). (LSM values partial out the effects of hen mass, cohort, period, personality in **b**, plus interactions). The legend denotes hens’ current housing during the time of testing. The pre-switch data are split by personality to show the interaction with housing treatment. ***P < 0.01, **P < 0.05, *P < 0.10.

A typical whole-body startle trace is shown in Fig. 5. We first analyzed the data using a repeated measures model to investigate within-subject effects across the housing switch. The effect of housing proved highly significant (F_1,35_ = 32.86, P < 0.001), suggesting that individual hens’ startle amplitudes were decreased by Enriched housing and increased by Control housing. However, interpretation of this within-subject result was complicated by a significant period*housing interaction, indicating that the magnitude of the effect depended on the order that hens received the housing treatments. To clarify this, between-subject analyses were conducted by splitting the data by housing period. The first of these analyses revealed that after the first five weeks of differential housing (i.e. pre housing switch) there was a housing*personality interaction (F_1,14_ = 14.30, P = 0.002). This interaction reflected a pattern somewhat similar to the judgment bias results: the housing effect was larger for Exploratory hens (F_1,6_ = 127.11, P < 0.001 [2679.23 ± 3308.31 mN versus 56768.58 ± 3701.50 mN]), than it was for Non-exploratory hens (F_1,7_ = 3.97, P = 0.087 [1093.54 ± 3373.33 mN versus 10647.63 ± 3373.33 mN]) (see Fig. 6a).

In the post-switch period, two weeks after housing treatments had been reversed, the housing*personality interaction was no longer significant (F_1,13_ = 0.19, P = 0.67), but the main effect of housing remained significant: hens now in Enriched housing (moved there from Control) now had significantly smaller startle amplitudes than hens now in Control housing (moved there from Enriched) (F_1,13_ = 6.85, P = 0.02 LSMs: −256.12 ± 4181.73 versus 15349.38 ± 3611.65]; Fig. 6b). Hen mass was also not a significant predictor of startle amplitude in any of these tests (P ≥ 0.21).

### Relationship between judgment bias and startle amplitude

Judgment bias did not predit startle amplitude. There was thus no main effect of proportion of probe lids flipped on startle (F_1,34_ = 0.125, P = 0.73). Nor were there any interactive effects, as would be expected if there was a relationship between the two measures in some sub-groups but not others (e.g. Proportion of lids flipped*housing: F_1,34_ = 0.202, P = 0.66; Proportion of lids flipped*personality: F_1,34_ = 0.57, P = 0.46); Proportion of lids flipped*housing*personality: F_1,34_ = 1.24, P = 0.27). The period*housing interaction also now had no significant effects on startle (P = 0.73).

### Sample size calculations

Startle amplitude proved to be a far more sensitive measure of housing’s welfare effects than judgment bias, regardless of whether personality was controlled for. Sample size estimates are provided in Table 2. (For judgment bias, Non-exploratory hens are omitted because their housing effect was not in the predicted direction).

**Table 2:** The estimated sample sizes required to detect a housing effect using between-subject t tests (with alpha = 0.05, and power = 0.8).

Subjects	Judgment bias	Startle amplitude
All hens (not controlling for personality)	164 per group	5 per group
Exploratory hens	85 per group	3 per group
Non-exploratory hens	N/A	4 per group

Estimates are based on the mean differences between housing treatments, pooled standard deviations and the resulting effect sizes (from the first tests run, before the housing switch; see Supporting Material), and were calculated using online calculators, e.g. http://www.sample-size.net/sample-size-means/. The “N/A” for Non-exploratory hens reflects the lack of predicted direction of the effect, thence lack of validity as an affect indicator, for judgement bias in this sub-group.

## Discussion

First, we revisit the assumptions and controls underpinning this experiment. Independent tests showed that Enriched environments containing resources *a priori* likely to be rewarding were indeed preferred by hens over Control pens (both after differential rearing for seven weeks and in the days following a housing switch). This supports our assumption that hens housed in the larger, more complex pens had more positive experiences, so inducing more positive affective states^3^. Like Asher *et al*. (2016)^57^, whose experiment on pigs this work was loosely modeled on, we would argue that such affective states were more positive moods. This is because “Emotional states occur in response to stimuli or situations that are actually, or potentially, rewarding or punishing”^3^, and because long-term mood states appear to reflect the moving average of recent emotional experiences^3,55,69^, such that, “a repeatedly rewarded animal will develop a more positive mood state that persists between individual rewards”^55^. Our affectively different housing systems thence provided us with a sound means of validating our two candidate welfare measures: startle amplitude, and responses in a task designed to assess judgment bias. Aside from assessing construct validity in this way, we also assessed our measures’ face validity: whether probe cues intermediate in colour to the training cues triggered intermediate responding (they did: darker shades of grey were treated decreasingly like the white DS+ cue and increasingly like the black DS− cue); and whether hens’ startle traces resembled those obtained for whole body reflexes in mammals (and again they did: the force plate successfully recorded traces lasting under 300 ms and resembling startle reflexes recorded in rats (^17,18^). Finally, we assessed personality, to parse out the variation this might introduce (since e.g. Asher *et al*. [2016] found that controlling for differences in personality improves judgment bias test sensitivity^57^. Our tests revealed reliable individual differences between hens, as found in other avian species^70^; and these personality differences did indeed contribute to variation in our welfare-relevant measures.

Turning to construct validation of the potential affect indicators, enriched housing did not consistently cause hens to respond more ‘optimistically’ in our colour-cued judgment bias tests. However, hens with Exploratory dispositions did show this response, so partially validating the task (for this subgroup only). Furthermore, such effects must have been quickly reversible: when we switched housing and re-assessed hens after only 10–12 days, Exploratory hens were now affected by their new housing in just the same way as they had been by their pre-switch housing. This is consistent with housing modulating hens’ affective states and changing mood, and suggests that just as affective states change during reward gain^71^ or loss^72^, so did any residual effects of past housing conditions quickly ‘washed out’ in our subjects.

Nevertheless, with its small effect size even in Exploratory birds (which would increase risks of Type II error in studies less well powered than our own), and lack of significant main effect across all hens (due to an even smaller overall effect size), our result joins several previous studies generating null or equivocal findings for animal judgment bias^5,6,73,74^), including work showing little influence of long-term differential housing on birds (starlings^66^; hens^75^; quails^76^) and published null findings in birds^63,66,75,77^. This could perhaps indicate that birds do not have strong judgment bias responses to affective changes (after all, in the few avian cases where predictions were partially supported, results may have been artefacts of study design, affectively unclear manipulations, or small sample sizes^59,60,62,78^); and/or, as outlined in the Introduction, that judgment bias tests are less sensitive to positive affect than to negative^8,52^. Such factors could even vary with personality: although subtle, our Exploratory hens did show the expected change in lid-flipping with housing, but the even smaller housing effect in Non-exploratory hens was in the opposite direction to that predicted. Together this highlights the need for more future work on the various factors that may influence the results of judgment bias tasks.

In contrast, our second potential measure of stimulus evaluation, startle amplitude, was much more sensitive to housing effects. Thus after five weeks in Enriched housing, hens exhibited large reductions in startle amplitudes across both personality types. Furthermore, this was not just a product of differential rearing having long-term developmental effects on, for instance, sensitivity to light: startle response patterns proved quickly reversible: when we switched housing and re-assessed startle reflexes after only 10–12 days, hens newly housed in Enriched housing now had smaller startle amplitudes than hens moved to Control housing. Again, like judgment bias effects in Exploratory hens, this is consistent with affective state changes rapidly induced by housing. Personality also modulated startle amplitude in the first phase of testing (although not following the housing switch). Thus at that time, housing affected Exploratory hens more than Non-exploratory hens: being raised in the smaller, relatively monotonous aversive Control pens impacted the former much more than the latter. One possible reason is that increased exploration in the personality tests reflected motivations to escape^5^ or to gain stimulation due to boredom-like states^79^, with Exploratory hens then being most prone to boredom or frustration in Control environments: a hypothesis for future test (see below). Such personality effects were not detectable after the housing swap, however, for reasons unknown (perhaps age blunts their impact, but more research would be needed to investigate this); and overall this means that unlike judgment bias, the effects of housing on startle were fairly consistent across hen personalities.

Together, this therefore provides the first evidence for affective modulation of startle in birds, and joins many results showing the potential of the startle reflex as an affect indicator in mammals (with useful properties that include being influenced bi-directionally by both positive and negatively valenced stimuli e.g.^19,21^). Our results also suggest that startle has great promise as a practical welfare assessment tool. Responses to housing showed very large effect sizes, meaning that effects would be detectable even with very small sample sizes (much smaller than those used here); and furthermore, assessing startle took only a fraction of the time needed to assess judgment bias (around four minutes total per hen, in contrast to the 6+ hours needed to train and test each hen in the judgment bias task). To date, some fear responses informally termed ‘startle responses’ have already been used in welfare research (e.g. retreating from a suddenly opened umbrella^80^; or stopping and looking at the source of a sudden sound^81^), but the true startle reflex – i.e. adirectional, and occurring within just a few hundred milliseconds of the eliciting stimulus^16–18^– has essentially not been utilized (see Kallnick *et al*.^82^ for a rare exception). We think this omission should be rectified, not just in chickens but in other species too. And next research steps for chickens should be to assess the specificity and sensitivity of their startle reflexes as indicators of affective state, to understand how and why this indicator may be prone to the false negatives and false positives reviewed in the Introduction.

Our third and final research aim was to test the hypothesis that judgment bias predicts startle, as would be expected if the two forms of stimulus evaluation share common underlying mechanisms, or if changes in judgment bias actually causes changes in startle. This is first time a study in animals has investigated whether these conceptually similar measures are empirically related. This hypothesis was not supported, however: decreased startle amplitude did not predict more positive judgment biases, not even in the Exploratory subset of hens in whom housing effects were most marked. Consequently, one possibility is that the two responses reflect independent processes. After all, despite both involving stimulus evaluation, they are quite different in nature. For one, judgment biases seem to be explicit effects: in humans, as outlined from the Introduction, they are generally inferred from cognitive tasks and verbal self-report, while in animals they are inferred from voluntary approach or avoidance behaviour which is assumed to involve executive cognitive control^62^. The startle reflex, in contrast, is implicit: too fast to be subject to intentional control^20^ (e.g. always unaffected by the direction of the eliciting stimulus). However, another explanation for the lack of correlation is that the methodologies for measuring startle and judgment biases diverge in terms of the hens’ experiences. For example, as typical for animal studies of this kind, our judgment bias task relied on extensive habituation, followed by discrimination training e.g.^52^ and the use of punishment and reward, with hens experiencing increasingly more reward as training progressed. These factors will all have influenced our subjects’ emotional states^71,83^; and by the end of training, hens should have learned that the judgment bias task yielded only reward (as long as the wrong lids were never flipped). In contrast, startle testing involved no habituation to the dark, unfamiliar and potentially anxiogenic chamber used, followed by the light flash itself (likely aversive, as reviewed in the Introduction): again, all factors likely to influence our subjects’ emotional states, but this time negatively. Such opposing effects on emotion in our two tests could, therefore, have added noise that masked any underlying relationships between the two responses. Future research should therefore replicate our work using judgment bias apparatuses that are rendered more aversive, and/or startle chambers rendered less so (perhaps via positive conditioning with mealworms), such that the affective impacts of the two tests themselves become more similar.

This idea thus adds to several other research avenues indicated by this experiment: the potential reasons for the weakness of our judgment bias effect, and its restriction to Exploratory hens; whether personality predicts boredom-like or frustrated states in different housing environments (*cf*.^79^); and assessment of the specificity and sensitivity of avian startle reflexes as indicators of more diverse affective states (e.g. those caused by acute reward, isolation, injuries and sickness). Furthermore, whether different testing methods have emotional impacts on research subjects that could mask (or exacerbate) the welfare states being investigated in them is also an important topic for welfare researchers. Finally, the practicalities of using methods other than force plates to assess startle (e.g. force transducers built into caging on farms) should also be investigated. But overall, startle reflex magnitudes emerge as a promising potential indicator of mood in laying hens: convenient and quick to assess, and far more sensitive than judgment bias at detecting the affective benefits of improved housing.

## Supplementary information

Supplementary Information

## Data Availability

The housing preference and startle data were analyzed using JMP (v. 13, SAS Institute, 2016), and the judgment bias data were analyzed with SAS 9.4 (SAS Institute Inc., Cary, NC, USA). The significance threshold was set at P < 0.05, and unless otherwise stated all tests were two-tailed to be conservative. To compare Enriched hens’ location preferences (Proxy Control pen versus Enriched pen), the relative difference in floor area was corrected for by multiplying the number of hens observed in the Proxy Control pen by a factor of six (the relative difference in floor area between environments) (see^53^ for more details). This adjusted estimate of the proportion of time hens spent in the Proxy Control pen was then tested against the hypothesis that hens occupied the Proxy Control and Enriched environments at chance levels using a one-tailed, one-sample Wilcoxon Signed Rank test. The url for archived data is: https://figshare.com/articles/SREP-19-04226A_Data_xlsx/9275582.
