# Supplementary material for: Welfare-improving enrichments greatly reduce hens’ startle responses, despite little change in judgment bias
Source: Sci Rep. 2019 Aug 15;9:11881. doi: 10.1038/s41598-019-48351-6 (PMC6695442; doi:10.1038/s41598-019-48351-6)
Supplement: Supplementary file 1 — Supplementary Information [file 41598_2019_48351_MOESM1_ESM.pdf]

Welfare-improving enrichments greatly reduce hens' startle responses, despite little change in judgment bias

Misha Ross, Anna Garland, Alexandra Harlander-Matauschek, Lindsey Kitchenham, and Georgia Mason

**Day**	**Procedure**
0	Move hens to experimental housing
0 - 33	Judgment bias task training
35, 37	Startle Reflex Test 1
38 - 42	Judgment Bias Test 1 (Sessions 1-5)
48	Switch housing
53 - 57	Judgment Bias Test 2 (Sessions 6 – 10)
58, 60	Startle Reflex Test 2

Table S1. Timeline showing the duration of housing treatments and order of testing.

**All hens**		**Mean**	**Stdev**	**N**	**Mean difference**	**Pooled SD**	**Cohen's D**
**JB**	Control	0.9354	0.2787	11	0.0745	0.2419	0.3081
	Enriched	1.0099	0.2028	12			
**Startle**	Control	4.2120	0.5482	11	-1.1214	0.4732	-2.370
	Enriched	3.0906	0.3929	12			

**Exploratory hens**		**Mean**	**Stdev**	**N**	**Mean difference**	**Pooled SD**	**Cohen's D**
**JB**	Control	0.9341	0.2785	5	0.1006	0.2348	0.4286
	Enriched	1.0347	0.1927	6			
**Startle**	Control	4.6587	0.1794	5	-1.4019	0.3827	-3.6628
	Enriched	3.2568	0.4878	6			

**Non-exploratory hens**		**Mean**	**Stdev**	**N**	**Mean difference**	**Pooled SD**	**Cohen's D**
**JB**	Control	0.9364	0.3054	6	0.04867	0.2694	0.1807
	Enriched	0.9851	0.2278	6			
**Startle**	Control	3.8397	0.4575	6	-0.9155	0.3497	-2.6182
	Enriched	2.9242	0.1877	6			

Table S2. Period 1 Effect size and pooled standard deviations for all hens, and for the two personality types
